# Supplementary material for: Rhs NADase effectors and their immunity proteins are exchangeable mediators of inter-bacterial competition in Serratia
Source: Nat Commun. 2023 Sep 28;14:6061. doi: 10.1038/s41467-023-41751-3 (PMC10539506; doi:10.1038/s41467-023-41751-3)
Supplement: Supplementary file 1 — Supplementary Information [file 41467_2023_41751_MOESM1_ESM.pdf]

# Supplementary Information

## Rhs NADase effectors and their immunity proteins are exchangeable mediators of inter-bacterial competition in *Serratia*

Martin Hagan[†], Genady Pankov[†], Ramses Gallegos-Monterrosa, David J. Williams, Christopher Earl, Grant Buchanan, William N. Hunter* & Sarah J. Coulthurst*

**Supplementary Figure 1.**

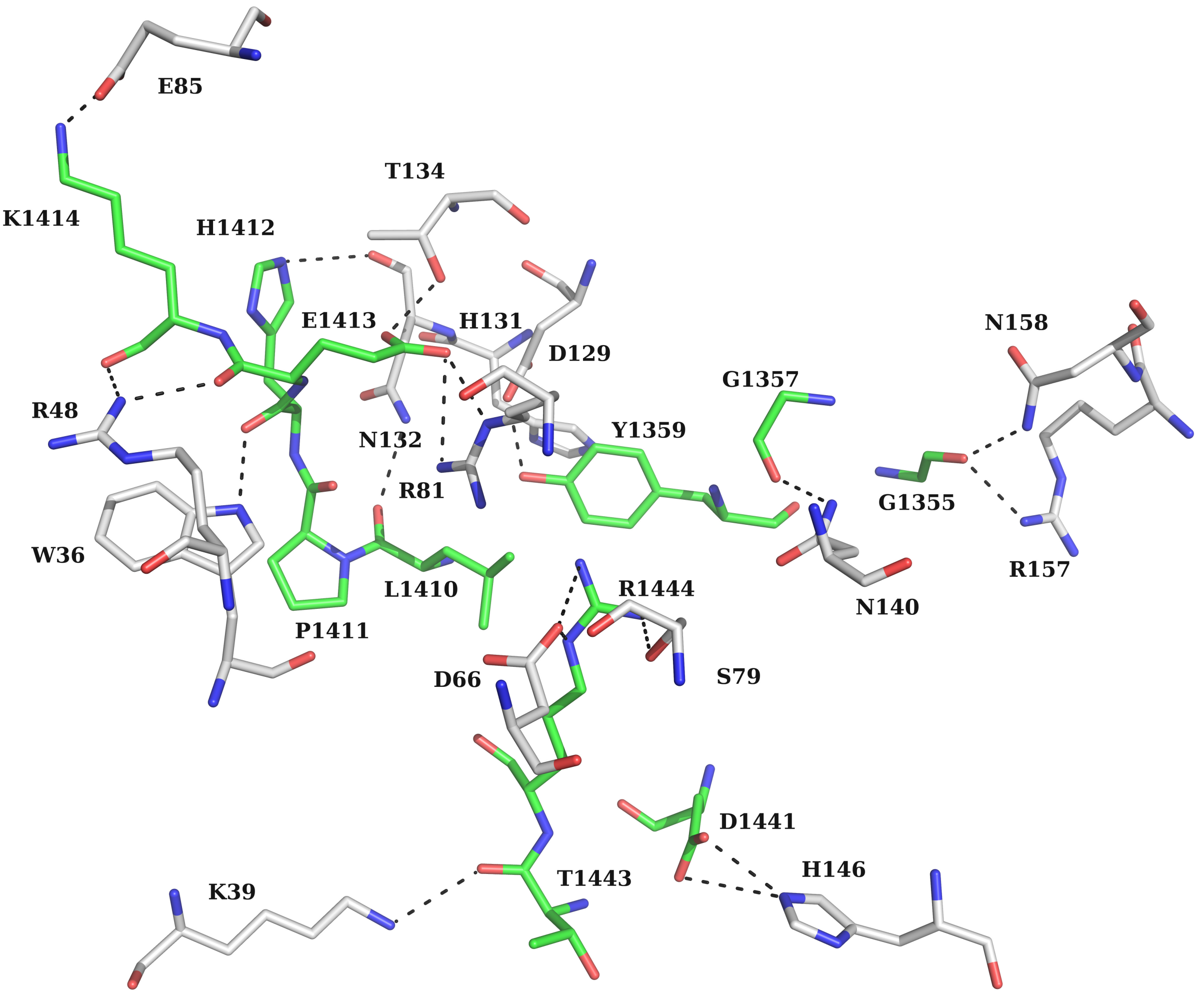


**Supplementary Figure 1. Residues involved in the formation of the protein-protein interface between Rhs1CT$_{Db10}$ and RhsI1$_{Db10}$.** Different coloring schemes were applied to the carbon atoms of Rhs1CT$_{Db10}$ (green) and RhsI1$_{Db10}$ (grey). Hydrogen bonds (within 2.5 Å–3.5 Å distance) are shown as dashed lines.

**Supplementary Figure 2.**

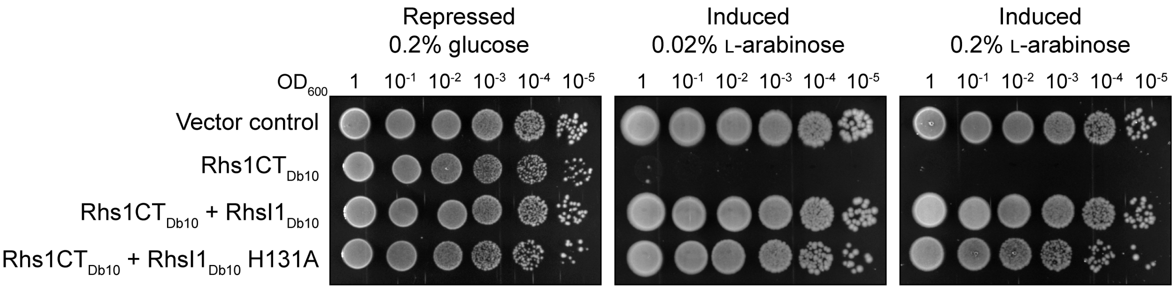


**Supplementary Figure 2. RhsI1$_{Db10}$ H131A retains the ability to neutralise the toxicity of Rhs1CT$_{Db10}$ when co-expressed in *E. coli*.** Growth of *E. coli* MG1655 carrying empty vector control plasmid (pBAD18-Kn) or plasmids directing the expression of wild type Rhs1CT$_{Db10}$ with N-terminal 3xFLAG tag alone (Rhs1CT$_{Db10}$), or Rhs1CT$_{Db10}$ together with wild type RhsI1$_{Db10}$ (+ RhsI1$_{Db10}$) or RhsI1$_{Db10}$ carrying a H131A substitution (+ RhsI1$_{Db10}$ H131A). Growth was on solid LB media with 0.2% glucose or 0.02% or 0.2% L-arabinose to repress or induce, respectively, gene expression. The data shown are representative of two independent experiments.

**Supplementary Figure 3.**

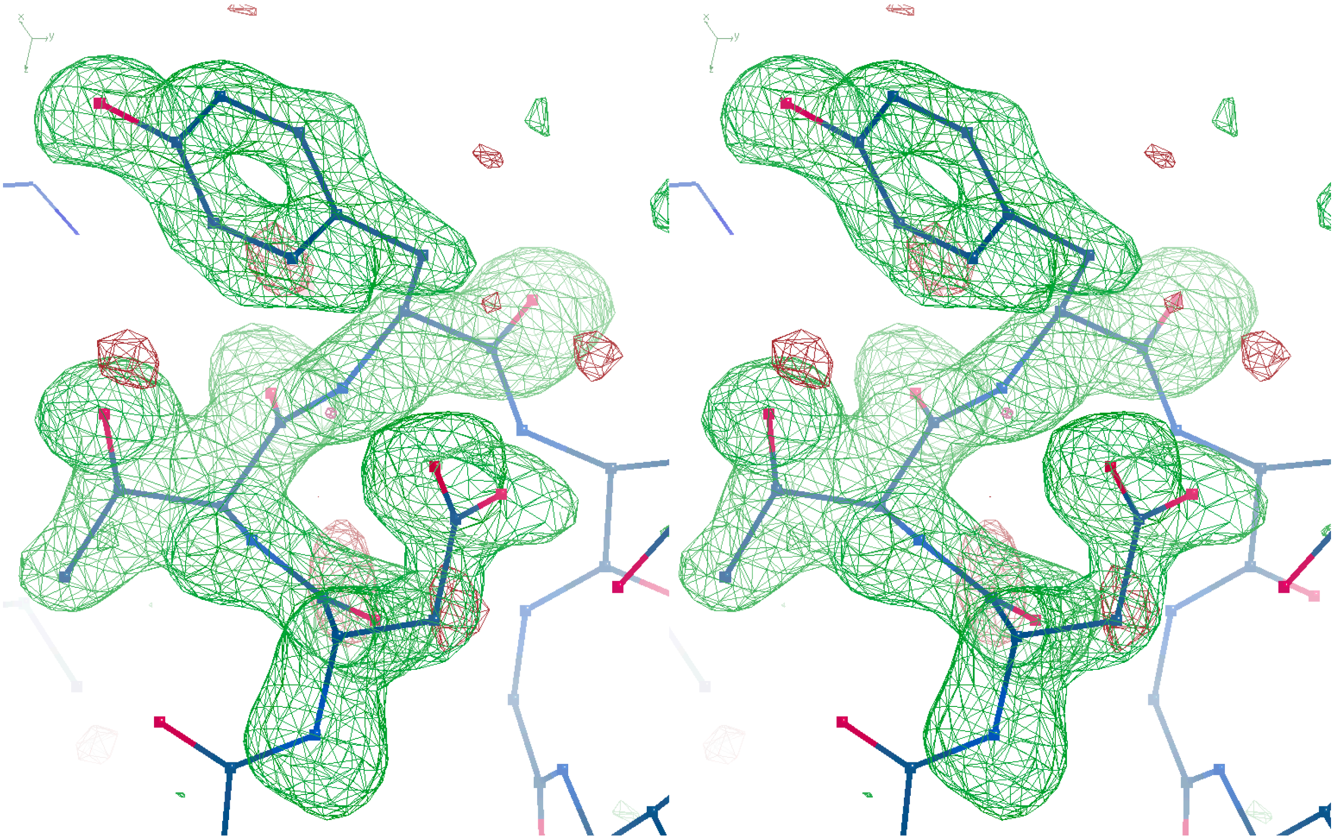

**Supplementary Figure 3. A side-by-side stereo view of a representative portion of the electron density map for the Rhs1CT$_{Db10}$-RhsI1$_{Db10}$ crystal structure (PDB 6XTD).** A fragment of the Fo-Fc omit map for RhsI1$_{Db10}$ with residues Asp4, Thr5 and Tyr6 contoured at 3σ level.

**Supplementary Table 1. Bacterial strains and plasmids used in this study.**

| Name | Description/genotype | Source / Reference |
|---|---|---|
| **Strains** | | |
| *Serratia marcescens* | | |
| Db10 | Wild type model strain | 1 |
| SJC1036 | Wild type clinical isolate | 2 |
| SJC11 | Db10 Δ*tssE* (*SMDB11_2271*) | 3 |
| FRC29 | Db10 Δ*rhs2*Δ*vgrG1* (*SMBD11_1610, SMBD11_2244*) | 4 |
| RGM32 | Db10 Rhs1_no CTI, Δ*rhs1CT*$_{Db10}$-*SMDB11_2281*. Lacks the region containing *rhs1CT*$_{Db10}$ (*SMDB11_2278* nt 3997-4422), *rhsI1 (SMDB11_2278A), SMDB11_2280* and *SMDB11_2281*. | This study |
| RGM33 | Db10 Rhs1_CTI$_{1036}$, *rhs1CT*$_{1036}$- *rhsI1*$_{1036}$ from *S. marcescens* SJC1036 replacing *rhs1CT*$_{Db10}$-*SMDB11_2281*. This generates a fusion of amino acids 1-1329 of Rhs1$_{Db10}$ with amino acids 1330-1486 of Rhs1$_{1036}$ (the exchanged region is the CT plus the final three amino acids of the core domain immediately prior to the cleavage site). | This study |
| RGM35 | Db10 Δ*tssE* Rhs1_CTI$_{1036}$, derived from SJC11 | This study |
| RGM36 | Db10 Δ*rhs2*Δ*vgrG1* Rhs1_CTI$_{1036}$, derived from FRC29 | This study |
| KT72 | Db10 rendered Sm$^R$ using φIF3 | 5 |
| JAD04 | Db10 Δ*2280*$_{Db10}$, in-frame deletion of *SMDB11_2280* | This study |
| JAD10 | JAD04 rendered Sm$^R$ using φIF3 | This study |
| *Escherichia coli* | | |
| MG1655 | Wild type (model K-12 strain) | 6 |
| BL21(DE3) pLysS | Protein overexpression strain. Chromosomal λDE3 encodes IPTG-inducible T7 RNA polymerase. pLysS directs expression of T7 lysozyme. | Novagen |
| CC118λ*pir* | Cloning host and donor strain for pKNG101-derived allelic exchange plasmids (λ*pir*) | 7 |
| HH26 pNJ5000 | Mobilizing strain for conjugal transfer | 8 |
| **Plasmids** | | |
| pRSFDuet-1 | Protein overexpression vector for the co-expression of two target genes. Each multiple cloning site (MCS) is preceded by a T7 promoter and the first site allows for fusion of an N-terminal His$_6$ tag (Kn$^R$) | Novagen |
| pET15b-TEV | Vector for protein overexpression under the control of the T7 promoter. Permits fusion of an His$_6$ tag followed by a TEV protease cleavage site to the N-terminus of the overexpressed protein (Ap$^R$) | 9 |
| pBAD18-Kn | Arabinose-inducible expression vector; gene of interest is cloned downstream of the P$_{ara}$ promoter (Kn$^R$) | 10 |
| pKNG101 | Suicide vector for allelic exchange (Sm$^R$, *sacBR, mobRK2, oriR6K*). | 11 |
| pSC962 | Coding sequence for His$_6$-Rhs1CT$_{Db10}$ (SMDB11_2278, amino acids 1333-1473) in MCS1 and untagged RhsI1$_{Db10}$ (SMDB11_2278A) in MCS2 of pRSFDuet-1. | This study |
| pSC981 | Coding sequence for His$_6$-Rhs1CT$_{1036}$ (SJC1036_02424, amino acids 1333-1486) in MCS1 and untagged RhsI1$_{1036}$ (SJC1036_02425) in MCS2 of pRSFDuet-1. | This study |
| pSC3020 | Coding sequence for Rhs1CT$_{1036}$ (SJC1036_02424, amino acids 1333-1486) in pET15b-TEV. The native, untagged protein is expressed. | This study |
| pSC3019 | Coding sequences for Rhs1CT$_{1036}$ (SJC1036_02424, amino acids 1333-1486) and RhsI1$_{1036}$ (SJC1036_02425) in pET15b-TEV. The native, untagged proteins are expressed. | This study |

| | | |
|---|---|---|
| pSC987 | Coding sequences for Rhs1CT$_{1036}$ (SJC1036_02424, amino acids 1333-1486) and 2280$_{Db10}$ (SMDB11_2280) in pET15b-TEV. The native, untagged proteins are expressed. | This study |
| pSC990 | Coding sequence for 3xFLAG-Rhs1CT$_{Db10}$ (SMDB11_2278; amino acids 1333-1473) in pBAD18-Kn | This study |
| pSC992 | Coding sequence for 3xFLAG-Rhs1CT$_{Db10}$ S1384A in pBAD18-Kn | This study |
| pSC993 | Coding sequence for 3xFLAG-Rhs1CT$_{Db10}$ S1399A in pBAD18-Kn | This study |
| pSC994 | Coding sequence for 3xFLAG-Rhs1CT$_{Db10}$ H1412A in pBAD18-Kn | This study |
| pSC999 | Coding sequence for 3xFLAG-Rhs1CT$_{Db10}$ F1386A in pBAD18-Kn | This study |
| pSC3500 | Coding sequence for 3xFLAG-Rhs1CT$_{Db10}$ R1418A in pBAD18-Kn | This study |
| pSC3501 | Coding sequence for 3xFLAG-Rhs1CT$_{Db10}$ Q1452A in pBAD18-Kn | This study |
| pSC4115 | Coding sequence for RhsI1$_{Db10}$ (SMDB11_2278A) in pSC990 | This study |
| pSC4116 | Coding sequence for RhsI1$_{Db10}$ (SMDB11_2278A) in pSC992 | This study |
| pSC4117 | Coding sequence for RhsI1$_{Db10}$ (SMDB11_2278A) in pSC993 | This study |
| pSC4118 | Coding sequence for RhsI1$_{Db10}$ (SMDB11_2278A) in pSC994 | This study |
| pSC4119 | Coding sequence for RhsI1$_{Db10}$ (SMDB11_2278A) in pSC999 | This study |
| pSC4120 | Coding sequence for RhsI1$_{Db10}$ (SMDB11_2278A) in pSC3500 | This study |
| pSC4121 | Coding sequence for RhsI1$_{Db10}$ (SMDB11_2278A) in pSC3501 | This study |
| pSC4112 | Coding sequence for RhsI1$_{Db10}$-HA (SMDB11_2278A) in pSC990 | This study |
| pSC4113 | Coding sequence for RhsI1$_{Db10}$-HA H131A in pSC990 | This study |
| pSC620 | pKNG101-derived allelic exchange plasmid for generation of an in-frame deletion of *SMDB11_2280*. | This study |
| pSC2776 | pKNG101-derived allelic exchange plasmid for deletion of the Db10 chromosomal region *rhs1CT$_{Db10}$* - *SMDB11_2281*. | This study |
| pSC2777 | pKNG101-derived allelic exchange plasmid for replacement of *rhs1CT$_{Db10}$* - *SMDB11_2281* on the chromosome of Db10 with *rhs1CT$_{1036}$-rhsI1$_{1036}$* from *S. marcescens* SJC1036 | This study |

## Supplementary Table 2. Oligonucleotide primers and synthetic gene fragments used for plasmid construction

| Plasmid | Sequence of relevant primers (5'-3')[a] | Description |
|---|---|---|
| pSC962 | TATAGGATCCGAAGCCACGATGCGCAGCAAC | Forward primer to clone SMDB11_2278 aa 1333-1473 in MCS1 of pRSFDuet-1 (*Bam*HI) |
| | TATA GAATTC TTACAATCCAATTTCAAAAGGC | Reverse primer to clone SMDB11_2278 aa 1333-1473 in MCS1 of pRSFDuet-1 (*Eco*RI) |
| | TATA CATATG ATGCAACTAGATACTTATGACG | Forward primer to clone SMDB11_2278A in MCS2 of pRSFDuet-1 (*Nde*I) |
| | TGTGCTCGAGTTAATAAATTATGTTATTCCTTTTGCAA TATTTTCG | Reverse primer to clone SMDB11_2278A in MCS2 of pRSFDuet-1 (*Xho*I) |
| pSC3020 | ATGCTCTAGACGAACACAGTGAACGATAGCCATGAGT TGCGGCAATTCCTGG | Forward primer to clone SJC1036_02424 aa 1333-1486 in pET15b-TEV (*Xba*I) |
| | ATGCGGATCCTCAAATTGGCCGGATTCG | Reverse primer to clone SJC1036_02424 aa 1333-1486 in pET15b-TEV (*Bam*HI) |
| pSC3019 | ATGCTCTAGACGAACACAGTGAACGATAGCCATGAGT TGCGGCAATTCCTGG | Forward primer to clone SJC1036_02424 aa 1333-1486 and SJC1036_02425 in pET15b-TEV (*Xba*I) |
| | ATGCGGATCCCTAGTGAAAAATGAAACCCAGC | Reverse primer to clone SJC1036_02424 aa 1333-1486 and SJC1036_02425 in pET15b-TEV (*Bam*HI) |
| pSC620 | TATAACTAGTGCGACAACCCGAGAAATGC | Forward primer to clone upstream region of SMDB11_2280 in pKNG101 (*Spe*I) |
| | GCGCAAGCTTCTCTTTGTTCATAATATTTAAATAGATC TAGTG | Reverse primer to clone upstream region of SMDB11_2280 in pKNG101 (*Hin*dIII) |
| | TGTGAAGCTTTTCATTTTTCACTAGCGCTCTCTTAGAG | Forward primer to clone downstream region of SMDB11_2280 in pKNG101 (*Hin*dIII) |
| | TATAGGGCCCTTGCTCGTCCATGGCATC | Reverse primer to clone downstream region of SMDB11_2280 in pKNG101 (*Apa*I) |
| pSC4115-pSC4121 | TATAGGTACCAAGAGGATTGTAAAATGCAAC | Forward primer to clone SMDB11_2278A into pSC990 and related plasmids (*Kpn*I) |
| | TATAGCATGCTTAATAAATTATGTTATTCC | Reverse primer to clone SMDB11_2278A into pSC990 and related plasmids (*Sph*I) |

| Plasmid | Details of synthetic insert |
|---|---|
| pSC981 | Synthetic insert including coding sequences for His$_6$-Rhs1CT$_{1036}$ and untagged RhsI1$_{1036}$, produced by GeneArt (ThermoFisher):<br>GGATCCAAGTTGCGGCAATTCCTGGAATAATTTTCAATCGAAGTCGAAAGGTGTTTTTGCATCCAGAAGCCAGGCATCTAA<br>AGCTTATAATCTGTGGAAAAAACAAGATTGGGCGGCCTTAGAAAAATTCATGGGGCATGGCTCATGGCCTCCCAATAGAG<br>GTTTTGTACAAGCAACGCCTACTACTCTTATGCCTGGAGCAAAAATTGATAGATACGGTGGCTGGACAAATAATGGCGTAT<br>TCAACGACCGAGGTACCTTTGTCTCTCCTGCTGGAGCCTCTTTTGGCAGCCGGGCATTACCGTTAGATACACTGGAAAAACC<br>GTATCGAGTATATGAGGTGGTTAAACCAATCCAAGCAGATATGGGGCCTGCTATACCGTGGTTTAACCAAGCTGGTGGAGG<br>AACACAATTTGAACTGTCCAAATCCATCAGTCAACTTCTTGCAGAAGGCCGAATCCGGCCAATTTGAGTCGACAAGCTTGC<br>GGCCGCATAATGCTTAAGTCGAACAGAAAGTAATCGTATTGTACACGGCCGCATAATCGAAATTAATACGACTCACTATAG<br>GGGAATTGTGAGCGGATAACAATTCCCCATCTTAGTATATTAGTTAAGTATAAGAAGGAGATATACATATGAGCGAAAAAT<br>ACGTTAGCGTGAAGAAAGATAAGCAACTGTTTGATGGTAACGAAGAGCAGTTTCACTTATTTAAAAAAGAAGTATCCATCC<br>CTAACTCTAACGAAAAAGTACTTTATGATAACAGCTCTGCGATACCCATATGGGATATAGATGAAAACATCAGGAAGGCG<br>ATCGCAGAAAAAATAAGCGGAATAAAAGTCATAGAGTATCCATATGACCCAATACCTTCATATAAAAATGAAAATCTGGA<br>TATCTATGTTCAACCAGTAGAGTCGGACATGCACAAAAAAAGAATAACAATAATCACGGCGACGGGTGAATCACAACCAG<br>CAAGATATAAGATAAAGTTGCTGGGTTTCATTTTTCACTAGCTCGAG |
| pSC987 | Synthetic insert containing coding sequences for the 3' end of Rhs1CT$_{1036}$ and 2280$_{Db10}$, used to replace RhsI1$_{1036}$ in pSC3019 with Db10$_{2280}$ in exactly the same context, produced by GeneArt (ThermoFisher):<br>GGTACCTTTGTCTCTCCTGCTGGAGCCTCTTTTGGCAGCCGGGCATTACCGTTAGATACACTGGAAAAACCGTATCGAGTAT<br>ATGAGGTGGTTAAACCAATCCAAGCAGATATGGGGCCTGCTATACCGTGGTTTAACCAAGCTGGTGGAGGAACACAATTTG<br>AACTGTCCAAATCCATCAGTCAACTTCTTGCAGAAGGCCGAATCCGGCCAATTTGAGGAATTATGAACAAAGAGAAAATTG<br>TCATTACCCCATCAAACAGGCAACTATTTGATGGTAACGAAGAGCAGTTTCGTTTATTTAAAAAAGAAGTATCCATCCCTA<br>ACTCTAACGAAAAAGTACTTTATGATAACAGCTCTGCGATACCCATATGGGATATAGATGAAAACATCAGGAAGGCGATC<br>GCAGAAAAAATAAGCGGGATAAAAGTCATAGAGTACCCATATGACCCAATACCTTCATATAAAAATGAAAATCTAGATAT<br>CTACATTCAGAATGTAGAGTCGGAAATGCACAAAAACAGAATAACAATAATCACGGCGACGGGTGAATCACAACCAGCAA<br>GATATAAGATAAAGTTGCTGGGTTTCATTTTTCACTAGGGATCC |
| pSC990 | Synthetic insert containing the coding sequence for parental Rhs1CT$_{Db10}$ (SMDB11_2278, amino acids 1333-1473) with an N-terminal 3xFLAG tag and a ribosome binding site for cloning into pBAD18-Kn, produced by GeneArt (ThermoFisher): |

| | |
|---|---|
| | GAGCTCAAGAGGAATACATATGGACTACAAAGACCATGACGGTGATTATAAAGATCATGATATCGATTACAAGGATGACG<br>ATGACAAAAAGCCACGATGCGCAGCAACCAAAGCCAACGATCATAATCAGGCAGCTTTTGGTCGACAATGGCAAGGTCGA<br>GGTATCTACAAAGGACGAGACTCTTGGTCAAATATCATGTTGAAAGAAGGTGACATTGTTTATGGCGGCGCTCCAGGGCAA<br>TCTGGTTTTTACTTCAACAAGGCGACGCTAGATGCTGCAGGTGGCAGTAGAGCCAAGCTATGGGAAAGTTTGCAGGTGCTT<br>CCACATGAAAAATTTGGTTATAGATCTAAAATACAAGCATATAGAGTAAAAAGAGAAACGATTGCAGGAACAGGTAAAGC<br>GATATCTCAAGACCCAACGAGATTCGGCGAAGGCGGAGGAACTCAATTTTTCCTTTCTAACTATAAAACTGTTCTTGAGCC<br>AATTGATAAGCCTTTTGAAATTGGATTGTAATCTAGAGGTACCGCATGC |
| pSC992 | Synthetic insert same as for pSC990 except for the incorporation of a S1384A mutation (tct→gct) |
| pSC993 | Synthetic insert same as for pSC990 except for the incorporation of a S1399A mutation (agt→gct) |
| pSC994 | Synthetic insert same as for pSC990 except for the incorporation of a H1412A mutation (cat→gcg) |
| pSC999 | Synthetic insert same as for pSC990 except for the incorporation of a F1386A mutation (ttt→gcg) |
| pSC3500 | Synthetic insert same as for pSC990 except for the incorporation of a R1418A mutation (aga→gct) |
| pSC3501 | Synthetic insert same as for pSC990 except for the incorporation of a Q1452A mutation (caa→gcg) |
| pSC4112 | Synthetic insert containing the coding sequence for parental RhsI1$_{\mathrm{Db10}}$ (SMDB11_2278A) with a C-terminal HA tag and a ribosome binding site for cloning into pSC990, produced by GenScript:<br>GGTACCAAGAGGATTGTAAAATGCAACTAGATACTTATGACGGAACACTAGAACTCGCGGGAATAACGCTAGGTACAGCG<br>ACAACCCGAGAAATGCTAATAAAAGGCTCCAGATTGTGGGAGGGTTGGCCAGAAAAGTCAGATGGTAGGACTACATCATA<br>CAGAACAATAATCAGTACAAAAAAAGAAAAAGCCGGCGATATATATATAATTGCCGACTTCTCCGGGGCCTTCATTACAG<br>ATGCAGTGCTTTGCAGTTGGCGTTTTGCGCCTGAAAAACTCATGATGGGAATACAAAAAAAAGTAGAAGGTGCTATTACCA<br>AAAATCTTCGAACATGGTTTTATGAAAAAACTCATATACAACTTCCAGTTAGTGGTTCATGGGGGCATATTGACGCTGCTTA<br>TGACCCACATAATTTAACGGGAACAATAGTGTGCAACTATCGCAGTGCATTTCATACTGAGGATGAATGGCGAAAATATTG<br>CAAAAGGAATAACATAATTTATGCCTATCCTTATGATGTTCCTGATTATGCATAAGCATGC |
| pSC4113 | Synthetic insert same as for pSC4112 except for the incorporation of a H131A mutation (cat→gcc) |
| pSC2776 | Synthetic insert for the deletion of *rhs1CT*$_{Db10}$ (*SMDB11_2278* nt 3997-4422) to *SMDB11_2281* (and the mutation of the final 10 nt of the core Rhs1CT to the corresponding bases in SJC1036, ttggggatgg→gttggggttg), flanked by 550 bp upstream and downstream sequence to facilitate allelic exchange by homologous recombination:<br>TCTAGAAAGCCCAGCAGCATGTGCATTTCCACTATGATCCGCTCGGGCGGCGCACGCAAAAGCGGGTGTGGCAGCAAAGT<br>CAGGATCTGCGCCAACCGGCCGGCAAATGCCAAACCACCACGTTTTTGTGGGAAGGTTTCCGGCTGCTGCAGGAAACTCGC<br>GACGGCATGCCCCTCACCTATGTCTATGCCGATCAGGGCAGCTATGAACCTTTGGCGCGCATCGACGGCCACGCGCCGGCC<br>CAAGTATTTTACTTCCACACGGCGCCGAACGGCGAACCGGAAAGCCTGACCGACAGCGACGGAACGCTGCGCTGGCAGAG<br>CCACAGCAGCGCCTGGGGCCGCATAAAGTATGAGGAAAATCAGCAAGATCTGGATTACTCCCAAAACCTGCGCCTGCAGG<br>GGCAATACCTGGATCGGGAAACGGGATTGCACTACAATTTGTTCCGCTATTACGATCCGGATATCGGCAGATTTACCCAGC<br>ACGATCCGATAGGTTTGGCCGGCGGGATCAACCTGTACCAATACGCGCCGAATCCGCTGGGTTGGGTGGATCCGTTGGGGT<br>TGTGAGAATTCACATGGAATAATTATCTTATGACTGTTCCTCTTTTAAATGAATCTTTAAATATCCCATCAAAAGGCTCCCA<br>AAGAGCCTTTTGATACGCTACCGCGCCGTCTCACCACTTCTTCACCGTCTGGTAAATCTCGAGGTCGAAATAGCCGTCGGCC<br>TCGCAGTCGTTGAGATAGTGCTCATAGCTCACCCCCTCAACCGGCTGGTAGCCGCTGGCCGGCAGCAGCTTCTGATAGAAC<br>TCGCCCCACACCTGCTCGAAATCGCCATCGCTGATGCGCACGTGGTAGACCGCATACTGCCCGGCGGGCAGCGTCTGCACG<br>GTCACGCCCTCGCTGCCCGCCGGCAGCGCGAAATCGTCCGCCACGCTTATCACTACGTCGGCACGCAGCTTTTCCGGCGCC<br>ACTTCCGCCGGATCGTCCCAATACAGCACCAGCCATTTGCCAAACGGCACGCCGTGGCGTTTCCGCCATGCCAGCAGTTGC<br>TGCGAGCCCTGCGGGATGGTCTGCGGATACGGCCCCACGACTCTCACTCCCACCACCTTCTCCGCCGCTTTATGGGCCC |
| pSC2777 | Synthetic insert for the replacement of *rhs1CT*$_{Db10}$ - *SMDB11_2281* with *rhs1CT*$_{1036}$-*rhsI1*$_{1036}$ (and the mutation of the final 10 nt of the core Rhs1CT to the corresponding bases in SJC1036, ttggggatgg→gttggggttg), flanked by 550 bp upstream and downstream sequence to facilitate allelic exchange by homologous recombination:<br>TCTAGAAAGCCCAGCAGCATGTGCATTTCCACTATGATCCGCTCGGGCGGCGCACGCAAAAGCGGGTGTGGCAGCAAAGT<br>CAGGATCTGCGCCAACCGGCCGGCAAATGCCAAACCACCACGTTTTTGTGGGAAGGTTTCCGGCTGCTGCAGGAAACTCGC<br>GACGGCATGCCCCTCACCTATGTCTATGCCGATCAGGGCAGCTATGAACCTTTGGCGCGCATCGACGGCCACGCGCCGGCC<br>CAAGTATTTTACTTCCACACGGCGCCGAACGGCGAACCGGAAAGCCTGACCGACAGCGACGGAACGCTGCGCTGGCAGAG<br>CCACAGCAGCGCCTGGGGCCGCATAAAGTATGAGGAAAATCAGCAAGATCTGGATTACTCCCAAAACCTGCGCCTGCAGG<br>GGCAATACCTGGATCGGGAAACGGGATTGCACTACAATTTGTTCCGCTATTACGATCCGGATATCGGCAGATTTACCCAGC<br>ACGATCCGATAGGTTTGGCCGGCGGGATCAACCTGTACCAATACGCGCCGAATCCGCTGGGTTGGGTGGATCCGTTGGGGT<br>TGAGTTGCGGCAATTCCTGGAATAATTTTCAATCGAAGTCGAAAGGTGTTTTTGCATCCAGAAGCCAGGCATCTAAAGCTT<br>ATAATCTGTGGAAAAAACAAGATTGGGCGGCCTTAGAAAAATTCATGGGGCATGGCTCATGGCCTCCCAATAGAGGTTTTG<br>TACAAGCAACGCCTACTACTCTTATGCCTGGAGCAAAAATTGATAGATACGGTGGCTGGACAAATAATGGCGTATTCAACG<br>ACCGAGGTACCTTTGTCTCTCCTGCTGGAGCCTCTTTTGGCAGCCGGGCATTACCGTTAGATACACTGGAAAAACCGTATCG<br>AGTATATGAGGTGGTTAAACCAATCCAAGCAGATATGGGGCCTGCTATACCGTGGTTTAACCAAGCTGGTGGAGGAACAC<br>AATTTGAACTGTCCAAATCCATCAGTCAACTTCTTGCAGAAGGCCGAATCCGGCCAATTTGAGGAATTATGAGCGAAAAAT<br>ACGTTAGCGTGAAGAAAGATAAGCAACTGTTTGATGGTAACGAAGAGCAGTTTCACTTATTTAAAAAAGAAGTATCCATCC<br>CTAACTCTAACGAAAAAGTACTTTATGATAACAGCTCTGCGATACCCATATGGGATATAGATGAAAACATCAGGAAGGCG<br>ATCGCAGAAAAAATAAGCGGAATAAAAGTCATAGAGTATCCATATGACCCAATACCTTCATATAAAAATGAAAATCTGGA<br>TATCTATGTTCAACCAGTAGAGTCGGACATGCACAAAAAAAGAATAACAATAATCACGGCGACGGGTGAATCACAACCAG<br>CAAGATATAAGATAAAGTTGCTGGGTTTCATTTTTCACTAGACATGGAATAATTATCTTATGACTGTTCCTCTTTTAAATGA<br>ATCTTTAAATATCCCATCAAAAGGCTCCCAAAGAGCCTTTTGATACGCTACCGCGCCGTCTCACCACTTCTTCACCGTCTGG<br>TAAATCTCGAGGTCGAAATAGCCGTCGGCCTCGCAGTCGTTGAGATAGTGCTCATAGCTCACCCCCTCAACCGGCTGGTAG<br>CCGCTGGCCGGCAGCAGCTTCTGATAGAACTCGCCCCACACCTGCTCGAAATCGCCATCGCTGATGCGCACGTGGTAGACC<br>GCATACTGCCCGGCGGGCAGCGTCTGCACGGTCACGCCCTCGCTGCCCGCCGGCAGCGCGAAATCGTCCGCCACGCTTATC |

| | |
|---|---|
| | ACTACGTCGGCACGCAGCTTTTCCGGCGCCACTTCCGCCGGATCGTCCCAATACAGCACCAGCCATTTGCCAAACGGCACG<br>CCGTGGCGTTTCCGCCATGCCAGCAGTTGCTGCGAGCCCTGCGGGATGGTCTGCGGATACGGCCCCACGACTCTCACTCCC<br>ACCACCTTCTCCGCCGCTTTATGGGCCC |

[a]Incorporated restriction sites for cloning into the respective vector are underlined.

**Supplementary Table 3. Crystallographic statistics for the Rhs1CT$_{Db10}$-RhsI1$_{Db10}$ complex**

| | |
|---|---|
| **Unit cell dimensions**<br>***a*, *b*, *c* [Å]**<br>***α*, *β*, *γ* [°]** | 39.66, 44.11, 46.81,<br>101.20, 96.13, 114.15 |
| **Space group** | *P*1 |
| **X-ray source** | Diamond Light Source, beamline I03 |
| **Wavelength [Å]** | 0.9150 |
| **Asymmetric unit** | heterodimer |
| **Resolution range** | 35.39-1.30 [1.32-1.30] |
| **Total number reflections** | 668801 [33385] |
| **Unique reflections** | 66880 [3188] |
| **Redundancy** | 10.2 [10.5] |
| $R_{merge}$[a] | 0.06 [1.2] |
| **Wilson *B* [Å$^2$]** | 14.3 |
| **Completeness [%]** | 96.1 [93.9] |
| **<I/σ[I]>** | 15.7 [2.1] |
| **CC[1/2]** | 1.00 [0.800] |
| $R_{pim}$[b] | 0.02 [0.40] |
| $R_{work}/R_{free}$[c] **[%]** | 12.2/16.7 |
| **Number reflections for** $R_{work}/R_{free}$ | 63553/3327 |
| **Protein residues** | 295 |
| **Ligands** | 6 bromides, 1 Bis-Tris |
| **Water molecules** | 392 |
| **R.M.S.D. Bonds [Å]/Angles [°]** | 0.013/1.608 |
| **Ramachandran plot** | |
| Residues in favored regions [%] | 98 |
| Residues in allowed regions [%] | 2 |
| Residues in outlier regions [%] | 0 |
| **Average *B*-factors**<br>**Protein atoms [Å$^2$]** | 18.3 Rhs1CT$_{Db10}$<br>20.1 RhsI1$_{Db10}$ |
| **Water molecules [Å$^2$]** | 33.2 |
| **Ligands of interest [Å$^2$]** | 19.2 |

*Numbers in parenthesis are for the highest resolution shell. Values in parentheses correspond to the highest resolution shell. [a.] $R_{merge} = \sum h \sum i |I[h,i] - \langle I[h]\rangle| / \sum h \sum i\, I[h,i]$; where I[*h,i*] is the intensity of the *i*th measurement of reflection *h* and <I[*h*]> is the mean value of I[*h,i*] for all *i* measurements.[b.] $R_{pim} = \sum h\ [1/[/nh-1]]^{1/2} \sum i|\langle Ih\rangle - Ih,i|/\sum h \sum i\ Ih,i$ [c.] $R_{work} = \sum hkl||F_o|-|F_c||/\sum|F_o|$, where $F_o$ is the observed structure factor amplitude and the $F_c$ is the structure-factor amplitude calculated from the model. $R_{free}$ is calculated with a subset of data that are excluded from refinement calculations (5 %) using the same method as for $R_{merge}$.

**Supplementary References**


1. Flyg C, Kenne K, Boman HG. Insect pathogenic properties of *Serratia marcescens*: phage-resistant mutants with a decreased resistance to *Cecropia* immunity and a decreased virulence to *Drosophila*. *Microbiology* **120**, 173-181 (1980).

2. Williams DJ*, et al.* The genus *Serratia* revisited by genomics. *Nature Communications* **13**, 5195 (2022).

3. Murdoch SL, Trunk K, English G, Fritsch MJ, Pourkarimi E, Coulthurst SJ. The opportunistic pathogen *Serratia marcescens* utilizes type VI secretion to target bacterial competitors. *Journal of Bacteriology* **193**, 6057-6069 (2011).

4. Cianfanelli FR, Alcoforado Diniz J, Guo M, De Cesare V, Trost M, Coulthurst SJ. VgrG and PAAR Proteins Define Distinct Versions of a Functional Type VI Secretion System. *PLoS Pathogens* **12**, e1005735 (2016).

5. English G, Trunk K, Rao VA, Srikannathasan V, Hunter WN, Coulthurst SJ. New secreted toxins and immunity proteins encoded within the Type VI secretion system gene cluster of *Serratia marcescens*. *Molecular Microbiology* **86**, 921-936 (2012).

6. Blattner FR*, et al.* The complete genome sequence of *Escherichia coli* K-12. *Science* **277**, 1453-1462 (1997).

7. Herrero M, de Lorenzo V, Timmis KN. Transposon vectors containing non-antibiotic resistance selection markers for cloning and stable chromosomal insertion of foreign genes in gram-negative bacteria. *Journal of Bacteriology* **172**, 6557-6567 (1990).

8. Grinter NJ. A broad-host-range cloning vector transposable to various replicons. *Gene* **21**, 133-143 (1983).

9. Rao VA, Shepherd SM, English G, Coulthurst SJ, Hunter WN. The structure of *Serratia marcescens* Lip, a membrane-bound component of the type VI secretion system. *Acta Crystallogr D Biol Crystallogr* **67**, 1065-1072 (2011).

10. Guzman L-M, Belin D, Carson MJ, Beckwith J. Tight regulation, modulation, and high-level expression by vectors containing the arabinose PBAD promoter. *Journal of Bacteriology* **177**, 4121-4130 (1995).

11. Kaniga K, Delor I, Cornelis GR. A wide-host-range suicide vector for improving reverse genetics in gram-negative bacteria: inactivation of the *blaA* gene of *Yersinia enterocolitica*. *Gene* **109**, 137-141 (1991).
