# Supplementary material for: Rhs NADase effectors and their immunity proteins are exchangeable mediators of inter-bacterial competition in Serratia
Source: Nat Commun. 2023 Sep 28;14:6061. doi: 10.1038/s41467-023-41751-3 (PMC10539506; doi:10.1038/s41467-023-41751-3)
Supplement: Supplementary file 2 — Reporting Summary [file 41467_2023_41751_MOESM2_ESM.pdf]

nature portfolio

Corresponding author(s): Sarah Coulthurst, Bill Hunter

Last updated by author(s): Aug 22, 2023

Reporting Summary

Nature Portfolio wishes to improve the reproducibility of the work that we publish. This form provides structure for consistency and transparency in reporting. For further information on Nature Portfolio policies, see our Editorial Policies and the Editorial Policy Checklist.

Statistics

For all statistical analyses, confirm that the following items are present in the figure legend, table legend, main text, or Methods section.

n/a	Confirmed	
☐	☒	The exact sample size (*n*) for each experimental group/condition, given as a discrete number and unit of measurement
☐	☒	A statement on whether measurements were taken from distinct samples or whether the same sample was measured repeatedly
☒	☐	The statistical test(s) used AND whether they are one- or two-sided<br>*Only common tests should be described solely by name; describe more complex techniques in the Methods section.*
☒	☐	A description of all covariates tested
☒	☐	A description of any assumptions or corrections, such as tests of normality and adjustment for multiple comparisons
☐	☒	A full description of the statistical parameters including central tendency (e.g. means) or other basic estimates (e.g. regression coefficient) AND variation (e.g. standard deviation) or associated estimates of uncertainty (e.g. confidence intervals)
☒	☐	For null hypothesis testing, the test statistic (e.g. *F*, *t*, *r*) with confidence intervals, effect sizes, degrees of freedom and *P* value noted<br>*Give P values as exact values whenever suitable.*
☒	☐	For Bayesian analysis, information on the choice of priors and Markov chain Monte Carlo settings
☒	☐	For hierarchical and complex designs, identification of the appropriate level for tests and full reporting of outcomes
☒	☐	Estimates of effect sizes (e.g. Cohen's *d*, Pearson's *r*), indicating how they were calculated

Our web collection on statistics for biologists contains articles on many of the points above.

Software and code

Policy information about availability of computer code

Data collection	- Crystallographic data were collected at beamline I03 equipped with an Eiger2 XE 16M detector at the Diamond Light Source (Didcot, UK).<br>- HPLC data were collected using UltiMate 3000 HPLC system equipped with Chromeleon (v6.8) software<br>- IMAC and SEC purification data were collected using ÄKTA pure system equipped with Unicorn 6.4 software<br>- Structural models were generated using AlphaFold2 provided by Google's Colab notebook service
Data analysis	- Crystallographic data were processed via the automated pipeline integrated in the XDS package. Data were scaled in Aimless, initial phases for bromide atoms were calculated using Crank2 and initial assignment of sequence to density performed using Buccaneer, all as part of the CCP4 v7.0 crystallographic package. Subsequent model building and refinement were performed using COOT and REFMAC5 within CCP4 v7.0 and MolProbity.<br>- Structural alignment was performed using COOT v0.9.6 (part of CCP4 v7.0 package).<br>- Further analysis of and comparison between structures was performed using Dali, XtalPred and PDBePISA.<br>- Molecular images were generated using PyMOL v2.5.2.<br>- Molar extinction coefficient was predicted using ProtParam<br>- Synteny plot was generated using genoPlotR v0.8.11 and R v4.0.3

For manuscripts utilizing custom algorithms or software that are central to the research but not yet described in published literature, software must be made available to editors and reviewers. We strongly encourage code deposition in a community repository (e.g. GitHub). See the Nature Portfolio guidelines for submitting code & software for further information.

Data

Policy information about availability of data

All manuscripts must include a data availability statement. This statement should provide the following information, where applicable:

- Accession codes, unique identifiers, or web links for publicly available datasets
- A description of any restrictions on data availability
- For clinical datasets or third party data, please ensure that the statement adheres to our policy

The coordinates and structure factors generated in this study have been deposited in the Protein Data Bank under accession code 6XTD [https://doi.org/10.2210/pdb6XTD/pdb]. All other data generated in this study are provided within the paper and its Supplementary Information files. Source Data are provided with this paper. Other structural data used in this study are available in the Protein Data Bank under accession codes 6B12 [https://doi.org/10.2210/pdb6B12/pdb] (Tne2CT-Tni2), 4ZV0 [https://doi.org/10.2210/pdb4ZV0/pdb] (Tse6CT-Tsi6), 6YGF [https://doi.org/10.2210/pdb6YGF/pdb] (AfNADase), 6YGG [https://doi.org/10.2210/pdb6YGG/pdb] (AfNADase in complex with benzamide adenine dinucleotide), and 4QLP [https://doi.org/10.2210/pdb4QLP/pdb] (TNT). Bacterial genome sequences used in this study have Genbank accession codes GCA_000513215.1 [https://www.ncbi.nlm.nih.gov/datasets/genome/GCF_000513215.1/] (Serratia marcescens Db11), GCA_946406795.1 [https://www.ncbi.nlm.nih.gov/datasets/genome/GCF_946406795.1/] (Serratia marcescens SJC1036) and GCA_003641105.1 [https://www.ncbi.nlm.nih.gov/datasets/genome/GCF_003641105.1/] (Serratia ficaria 1D1416).

Human research participants

Policy information about studies involving human research participants and Sex and Gender in Research.

Reporting on sex and gender	N/A
Population characteristics	N/A
Recruitment	N/A
Ethics oversight	N/A

Note that full information on the approval of the study protocol must also be provided in the manuscript.

Field-specific reporting

Please select the one below that is the best fit for your research. If you are not sure, read the appropriate sections before making your selection.

☒ Life sciences ☐ Behavioural & social sciences ☐ Ecological, evolutionary & environmental sciences

For a reference copy of the document with all sections, see nature.com/documents/nr-reporting-summary-flat.pdf

Life sciences study design

All studies must disclose on these points even when the disclosure is negative.

Sample size	No sample size calculations were performed. Four independent biological replicates were performed for the antibacterial activity assay since previous work in our laboratory and the literature indicated that this is sufficient to detect a substantive difference between different strains being tested in such an assay.
Data exclusions	There were no data exclusions.
Replication	Attempts at replication were successful. Antibacterial assays were performed with four independent biological replicates. All plate-based toxicity assays and western blots were performed on at least two independent occasions with biologically independent samples, with very similar results on each occasion. NADase activity was observed using two independent preparations of Rhs1CTDb10.
Randomization	No randomisation was performed. This was an in vitro study which did not involve human participants or animal models and thus randomisation was not required.
Blinding	No blinding was performed. Blinding is not applicable for the in vitro approaches used in the study (data values are not derived from subjective observations, nor are human or animal subjects interacting with investigators).

Reporting for specific materials, systems and methods

We require information from authors about some types of materials, experimental systems and methods used in many studies. Here, indicate whether each material, system or method listed is relevant to your study. If you are not sure if a list item applies to your research, read the appropriate section before selecting a response.

Materials & experimental systems

n/a	Involved in the study
☐	☒ Antibodies
☒	☐ Eukaryotic cell lines
☒	☐ Palaeontology and archaeology
☒	☐ Animals and other organisms
☒	☐ Clinical data
☒	☐ Dual use research of concern

Methods

n/a	Involved in the study
☒	☐ ChIP-seq
☒	☐ Flow cytometry
☒	☐ MRI-based neuroimaging

Antibodies

Antibodies used

Monoclonal anti-FLAG (Mouse, clone M2, raised against DYKDDDDK, #F3165, Sigma).
Horse radish peroxidase (HRP)-conjugated goat anti-mouse (#170-6516, Bio-Rad).

Validation

Both are commercial antibodies used routinely in E. coli and other Gram-negative bacteria. Previous work in our lab and the negative vector-only controls in Figure 5b confirm the specificity of the antibody for the detection of the fusion protein of interest.
